# Test-Retest of the Spot Vision Screener among Children with Ophthalmological Diseases including Strabismus

**DOI:** 10.1155/2024/2173860

**Published:** 2024-05-06

**Authors:** Mika Ichimura, Satoshi Ueki, Takeo Fukuchi

**Affiliations:** Division of Ophthalmology and Visual Science, Graduate School of Medical and Dental Sciences, Niigata University, Niigata, Japan

## Abstract

**Background:**

The spot vision screener (SVS) has been widely used for eye health examinations of infants and young children. The purpose of this study was to evaluate the reproducibility of two SVS measurements in children with ophthalmological diseases.

**Methods:**

29 patients aged 15 years or younger who visited our hospital for refraction examinations with SVS before and at least 60 minutes after administration of 2 drops of 1% cyclopentolate ophthalmic solution (before and after cycloplegia) were included in this study. Two SVS measurements were made before and after cycloplegia, respectively. Intraclass correlation coefficients (ICCs) and Bland–Altman analysis for spherical, spherical equivalent (SE), cylindrical, J0, and J45 values before and after cycloplegia were analyzed.

**Results:**

The mean age ± standard deviation (SD) of the 29 patients was 7.6 ± 2.4 years. There were 11 males and 18 females. The mean spherical values based on the SVS before and after cycloplegia were 0.42 ± 1.67 diopter (D), and 1.47 ± 2.23 D for the first measurement and 0.60 ± 1.74 D, and 1.42 ± 2.27 D for the second measurement, respectively. The mean cylindrical values based on SVS before and after cycloplegia were −1.45 ± 0.96 D and −1.65 ± 0.89 D for the first measurement and −1.58 ± 1.13 D and −1.66 ± 0.91 D for the second measurement, respectively. The ICCs for the first and second spherical, SE, cylindrical, J0, and J45 values before cycloplegia were 0.95, 0.98, 0.83, 0.86, and 0.86, respectively. The ICCs for the first and second spherical, SE, cylindrical, J0, and J45 values after cycloplegia were 0.99, 0.99, 0,87, 0.73, and 0.80, respectively. The Bland–Altman analysis of the first and second spherical and SE values before cycloplegia showed fan-shaped variation as hyperopia increased.

**Conclusions:**

Two consecutive SVS refraction measurements have a high degree of reproducibility for spherical and SE values but a low degree for cylindrical, J0, and J45 values. From these results, multiple measurements are required to obtain reliable results for cylindrical values.

## 1. Background

The spot vision screener (SVS) (Welch Allyn, Skaneateles Falls, NY, USA) is a refractive measurement device based on photorefraction. Since the SVS is easy and quick to use and has a high measurement availability rate, it has been widely used for eye health examinations of infants and young children [1–3]. Many studies have examined the measurement accuracy of the SVS in comparison with other existing refractive measurement devices [4–7]. Since the measurement results displayed by the SVS are only one-time measurement values, many reports use the average of several measurement results for analysis [4–6, 8]. In order to introduce the SVS more widely to eye health examinations, it is important to determine how many measurements are needed to accurately measure refraction. The purpose of this study was to evaluate the reproducibility of the SVS by measuring refraction twice in children. In addition, since the SVS measurement method is based on photorefraction, we hypothesized that pupil diameter would have a strong influence on the measurement results. Thus, we conducted two measurements each under two different conditions, with or without a cycloplegic agent. There are three portable photoscreening autorefractometers commercially available. Arnold et al. have reported the instrument referral criteria for the three, i.e., SVS, PlusoptiX (Nuremberg, Germany), and 2WIN (Padova, Italy) [9]. Although these share infrared and eccentric flash mechanism, the manufacturers' software differ including time and design features favoring a quick, child-friendly method versus refractive accuracy and precision.

## 2. Methods

Of the patients with ophthalmological diseases aged 15 years or below who visited the Department of Ophthalmology, Niigata University Medical and Dental Hospital between February 2020 and October 2020 for refraction examinations by SVS (the software version was 3.1.00.00-A004) before and at least 60 minutes after administration of 2 drops of 1% cyclopentolate ophthalmic solution over a 5 minute interval (before and after cycloplegia) were included in the study. Because refractive values in the right eye were analyzed in this study, patients who had retinal or optic nerve disease in the right eye were excluded. Eventually, 29 patients were included in this study. The following characteristics of the 29 patients were retrospectively analyzed: age, sex, best corrected visual acuity measured with Landolt chart, and refractive values measured using the SVS before and after cycloplegia. SVS measurements were made twice before and after cycloplegia, respectively. Our intent was not to compare the noncycloplegic to the cycloplegic SVS refractions for the right eyes. We did not compare values measured by SVS to those measured by autorefractometer, the gold standard.

SVS measurements were made in a semidarkroom. Both eyes were measured simultaneously in patients without strabismus. In patients with strabismus, one eye was shielded by the hand of the patient or their guardian. Five optometrists were in charge of the examinations. Each optometrist asked the patient to keep the face from tilting and to fixate on the SVS during the examination.

This retrospective study was approved by the Institutional Review Board/Ethics Committee of Niigata University (registration number, 2019-0094). It followed the tenets of the Declaration of Helsinki. Informed consent was obtained using the opt-out method because of the study's retrospective nature. None of the parents or guardians of the patients included signed and returned the opt-out form.

## 3. Statistical Analysis

To analyze reproducibility of two consecutive refractive values, refractive measurements of the right eye were analyzed. Intraclass correlation coefficients (ICCs) for spherical, spherical equivalent (SE), cylindrical, J0, and J45 values before and after cycloplegia were analyzed with IBM SPSS Statistics 25 (IBM Corp, Armonk, NY, USA). Positive values of J0 indicate with-the-rule astigmatism and negative values of J0 indicate against-the-rule astigmatism [10]. J45 represents oblique astigmatism [10]. Before the ICC analysis, paired *t*-tests were performed to compare each refractive parameter between 1st and 2nd measurements with SigmaPlot 14 (Systat Software, San Jose, CA, USA). A *p* value <0.05 was considered statistically significant. The Bland–Altman analysis was performed with SigmaPlot 14.

To analyze variations across ages and genders, Pearson' s correlation to analyze correlations between differences in each refractive parameter and age, and unpaired *t*-tests to compare each refractive parameter between male and female were performed with SigmaPlot 14 (a *p* value <0.05 was considered statistically significant).

## 4. Results

The mean age ± standard deviation (SD) of the 29 patients was 7.6 ± 2.4 years (3–11 years). There were 11 males and 18 females. Ophthalmological diseases were strabismus in 17 patients (exotropia in 11 patients, esotropia in 5 patients, and congenital superior oblique palsy in 1 patient), amblyopia in 10 patients, and congenital ptosis in 4 patients. 1 patient had exotropia and amblyopia both. Another patient had exotropia and congenital ptosis both. Of the 11 patients with exotropia, 7 had constant exotropia, 3 had intermittent exotropia, and 1 had consecutive exotropia. Decimal visual acuity ranged from 0.6 to 1.0, with 1.0 in 23 patients, 0.9 in 2 patients, 0.8 in 2 patients, 0.7 in 1 patient, and 0.6 in 1 patient.

The mean spherical values based on the SVS before cycloplegia were 0.42 ± 1.67 diopter (D) (−3.5–4.0 D) for the first measurement and 0.60 ± 1.74 D (−3.75–3.75 D) for the second measurement. The mean spherical values based on the SVS after cycloplegia were 1.47 ± 2.23 D (−3.75–7.25 D) for the first measurement and 1.42 ± 2.27 D (−3.5–7.5 D) for the second measurement. The mean cylindrical values based on SVS before cycloplegia were −1.45 ± 0.96 D (−3.75–0 D) for the first measurement and −1.58 ± 1.13 D (−3.75–0 D) for the second measurement. The mean cylindrical values based on SVS after cycloplegia were −1.65 ± 0.89 D (−3.25–−0.25 D) for the first measurement and −1.66 ± 0.91 D (−3.5––0.25 D) for the second measurement. The spherical, SE, cylindrical, J0, and J45 values before and after cycloplegia are shown in Tables 1 and 2. There were no statistically significant differences with paired *t*-tests for each refractive parameter between 1st and 2nd measurements.

The ICC results are shown in Tables 3 and 4. The ICC for the first and second spherical, SE, cylindrical, J0, and J45 values before cycloplegia was 0.95, 0.98, 0.83, 0.86, and 0.86, respectively. The ICC for the first and second spherical, SE, cylindrical, J0, and J45 values after cycloplegia was 0.99, 0.99, 0.87, 0.73, and 0.80, respectively.

**Figure 1:**
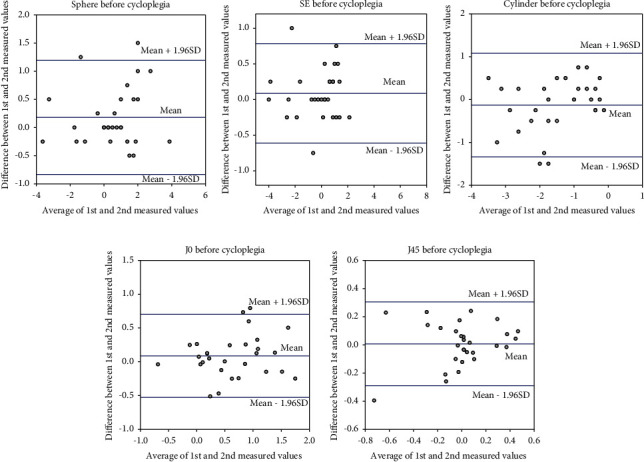
Bland–Altman plot for each refractive parameter before cycloplegia: (a) first and second spherical values. The plot shows fan-shaped variation as hyperopia increased; two patients were beyond 1.96 standard deviations (SDs). (b) first and second spherical equivalent (SE) values. The plot shows fan-shaped variation as hyperopia increased; two patients were beyond 1.96 SDs. (c) first and second cylindrical values. The plot shows that two patients were beyond 1.96 SDs. (d) first and second J0 values. The plot shows that two patients were beyond 1.96 SDs. (e) first and second J45 values. The plot shows that one patient was beyond 1.96 SDs.

**Figure 2:**
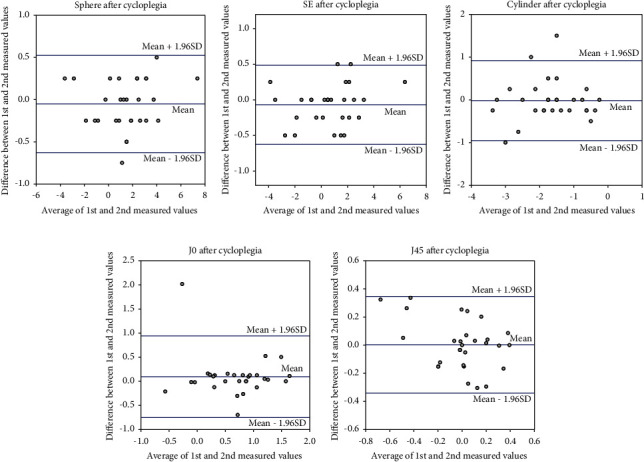
Bland–Altman plot for each refractive parameter after cycloplegia: (a) first and second spherical values. The plot shows that one patient was beyond 1.96 standard deviations (SDs). (b) first and second spherical equivalent (SE) values. The plot shows that two patients were beyond 1.96 SDs. (c) first and second cylindrical values. The plot shows that three patients were beyond 1.96 SDs. (d) first and second J0 values. The plot shows that one patient was beyond 1.96 SDs. (e) first and second J0 values. The plot shows that there was no patient beyond 1.96 SDs.

The Bland–Altman analysis results are shown in Figures 1 and 2. There was a fan-shaped distribution of the first and second spherical and SE values before cycloplegia as hyperopia increased; two patients were beyond 1.96 SDs, respectively. In the Bland–Altman analysis of the first and second cylindrical, J0, and J45 values before cycloplegia, two patients were beyond 1.96 SDs in cylindrical and J0 values, and one patient in J45 values. The respective Bland–Altman analyses of the first and second spherical and SE values after cycloplegia showed that one patient and two patients were beyond 1.96 SDs, respectively. The respective Bland–Altman analyses of the first and second and cylindrical, J0, and J45 values after cycloplegia showed that three patients, one patient, and no patient was beyond 1.96 SDs, respectively. There were three patients beyond 1.96 SD on two parameters, and one patient beyond 1.96 SD on four parameters (sphere after cycloplegia, cylinder before and after cycloplegia, and J0 before cycloplegia). The patient beyond 1.96 SD on four parameters had exotropia and a history of autism spectrum disorder.

Pearson's correlation revealed no statistically significant correlations between differences in each refractive parameter and age. Unpaired *t*-tests revealed no statistically significant differences for each refractive parameter between males and females.

## 5. Discussion

In this study of the reproducibility of two consecutive SVS refractive value measurements before cycloplegia, the ICC for spherical, SE, cylindrical, J0, and J45 values were 0.95, 0.98, 0.83, 0.86, and 0.86, respectively. The reproducibility of two consecutive refractive value measurements by the SVS for sphere and SE was good, whereas that for cylinder, J0, and J45 was relatively poor. These results are most likely due to the fact that the head tilt could not be strictly corrected in this study although each optometrist asked the patient to keep the face from tilting and to fixate on the SVS during the examination. In addition, in this study, five optometrists performed refractive value measurements with SVS. The problem of interexaminer measurement error remains. Considering the results of this study, multiple measurements are required to obtain reliable results for cylindrical values. The Bland–Altman analysis showed that spherical and SE values varied in a fan shape as hyperopia increased, suggesting the existence of a proportional error.

In this study, we also analyzed the reproducibility of two consecutive SVS refractive measurements after cycloplegia. The ICCs for spherical and SE, after cycloplegia, were 0.99 for both, higher than ICCs before cycloplegia. The Bland–Altman analysis showed less variability in spherical and SE values. The fan-shaped variations associated with hyperopia, before cycloplegia, were also absent. Although SVS measurements are considered to be less prone to accommodation because of the 1 m distance measurement, the difference between these results before and after cycloplegia is attributable to decreased accommodation and dilated pupils after cycloplegia. Yakar analyzed the sensitivity and specificity of the SVS for detecting refractive errors before and after cycloplegia in children aged 3–10 years [11]; cycloplegia improved sensitivity and negative predictive values. It may be necessary to use cycloplegic eye drops in patients who require full correction. Peterseim et al. have reported that refractive values measured with the SVS are underestimated in patients with high hyperopia in the absence of cycloplegia [12].

The ICCs for cylindrical, J0, and J45 values after cycloplegia were 0.87, 0.73, and 0.80, respectively. For J0 and J45 values, ICCs after cycloplegia were poorer than those before cycloplegia. It has been reported that with-the-rule astigmatism and against-the-rule astigmatism increases after cycloplegia [13]. Asharlous et al. have proposed several reasons for that astigmatism increase after cycloplegia, accommodative astigmatism, subject's head positioning, high order aberration increment, and different centrifugal astigmatism vectors in different centrifugal rings [13]. They consider that accommodative astigmatism is the main cause [13]. Since this study underwent refractive measurement before and after cycloplegia on the same day, it is possible that subject's fatigue due to waiting time could have affected subject's head positioning.

Variations across ages and genders were not observed, although patients included in this study were a small number.

This study found that two consecutive SVS refraction measurements have a high degree of reproducibility for spherical and SE values, whereas that for cylindrical, J0, and J45 values were relatively poor. To prevent the subject's face from tilting and encourage the subject to fixate their gaze is probably important. However, in children, it is difficult to hold the head position strictly by encouraging alone, which may have affected the astigmatism measurement. Furthermore, this study revealed that SVS was allowed to compare monocular refraction. The internal interpretation paradigm in SVS may favor speed and child-friendliness at the expense of astigmatism precision. Future studies with younger age groups to evaluate whether SVS can be more widely used for ophthalmic health examinations of infants and young children are needed.

## Figures and Tables

**Table 1:** The spherical, SE, cylindrical, J0, and J45 values (diopter) before cycloplegia.

	Mean ± SD	Minimum	Maximum
Spherical values 1st	0.42 ± 1.67	−3.5	4
Spherical values 2nd	0.60 ± 1.74	−3.75	3.75
SE values 1st	−0.26 ± 1.61	−4.0	2.25
SE values 2nd	−0.17 ± 1.62	−4.0	2.0
Cylindrical values 1st	−1.45 ± 0.96	−3.75	0
Cylindrical values 2nd	−1.58 ± 1.13	−3.75	0
J0 values 1st	0.60 ± 0.57	−0.67	1.87
J0 values 2nd	0.69 ± 0.62	−0.71	1.88
J45 values 1st	0.00 ± 0.27	−0.74	0.42
J45 values 2nd	0.01 ± 0.30	−0.92	0.51

SD: standard deviation; SE: spherical equivalent.

**Table 2:** The spherical, SE, cylindrical, J0, and J45 values (diopter) after cycloplegia.

	Mean ± SD	Minimum	Maximum
Spherical values 1st	1.47 ± 2.23	−3.75	7.25
Spherical values 2nd	1.42 ± 2.27	−3.5	7.5
SE values 1st	0.64 ± 2.16	−4.0	6.25
SE values 2nd	0.57 ± 2.22	−3.75	6.5
Cylindrical values 1st	−1.65 ± 0.89	−3.25	−0.25
Cylindrical values 2nd	−1.66 ± 0.91	−3.5	−0.25
J0 values 1st	0.64 ± 0.62	−1.27	1.59
J0 values 2nd	0.74 ± 0.56	−0.67	1.75
J45 values 1st	−0.01 ± 0.31	−0.84	0.43
J45 values 2nd	−0.00 ± 0.24	−0.52	0.42

SD: standard deviation; SE: spherical equivalent.

**Table 3:** ICCs for SE and cylindrical values before cycloplegia.

	ICC (1, 1)	95% confidence interval	
Spherical values	0.95	0.90	0.98
SE values	0.98	0.95	0.99
Cylindrical values	0.83	0.67	0.91
J0 values	0.86	0.72	0.93
J45 values	0.86	0.72	0.93

ICC: intraclass correlation coefficient; SE: spherical equivalent.

**Table 4:** ICCs for SE and cylindrical values after cycloplegia.

	ICC (1, 1)	95% confidence interval	
Spherical values	0.99	0.98	1.00
SE values	0.99	0.98	1.00
Cylindrical values	0.87	0.73	0.93
J0 values	0.73	0.50	0.86
J45 values	0.80	0.63	0.90

ICC: intraclass correlation coefficient; SE: spherical equivalent.

## Data Availability

The data used to support the findings of this study are available from the corresponding author upon reasonable request and are included within the article.

## Abbreviations

SVS: Spot vision screener
ICC: Intraclass correlation coefficient
SD: Standard deviation
D: Diopter
SE: Spherical equivalent.
